# Supplementary material for: Factors That Support Technology Integration Practices in the Math Education of Children with Intellectual Disabilities
Source: Children (Basel). 2023 May 23;10(6):918. doi: 10.3390/children10060918 (PMC10296979; doi:10.3390/children10060918)
Supplement: Supplementary file 1 [file children-10-00918-s001.zip › children-2236079-supplementary.pdf]

Supplementary material

A. Content of the questionnaire:

Technology integration practices

P1. What are the types of digital technologies you use in Mathematics activities?

P2. In general, what would be a mean percent from a lessons's time in which you used digital technology? Please refer to the current year.

P3. In most situations, estimate a mean percentage of the taught content in which you used technology. Please refer to the current year.

P4. During online school I have used the following digital technologies to teach Math.

P5. When returning from online school to on- site, I have used the following technologies:

P6. Underneath, you have a list of curricular math topics that you address with your students with intellectual disabilities. Give examples of digital technologies that you used to teach various content. You can include an example. (Math prerequisites/ Number concept and numeracy/ Addition, subtraction, multiplication, division/ Word problem solving (simple, complex, algorithmic)/ Measurement units/ Elements of geometry)

P7. For which teaching function have you predominantly used digital technologies?

Exercise/ Teaching of new concepts/ Assessment/ I do not use it

P8. Select educational digital resources that you use in your Math activities: digital textbook, web pages, encyclopedias, videos.

P9. What type of emergent technologies have you use in the classroom?

None/Augmented reality/ Virtual reality/ Robotics/ Other

P10. From the following educational digital resources, indicate the ones that you most created with available apps: electronic presentations/ virtual panels/ videos, animation, animated strips/ collaborative documents/ conceptual maps/ educational games and interactive exercises/ assessment instruments.

Technology related knowledge items

TRK1. Which technology would you recommend being used in the Math education of children with intellectual disabilities on a large scale?

TRK2. Enumerate some advantages of using mobile Math games in the Math education of children with intellectual disabilities.

TRK3. Enumerate some advantages of using interactive PowerPoint in the Math education of children with intellectual disabilities.

TRK4. Underneath, you have possible uses of technology in Math education. Give an example of technology that can support

TRK4a. the formation of Mathematical representations.

TRK4b. discovery learning

TRK4c. individualization of learning

TRK4d. independent work and exercise

TRK5. Do you know technologies of Augmented reality?

TRK6. Give examples of apps/ software that allow you to develop digital resources for Math, such as virtual recapitulative panels.

TRK7. Give examples of apps/ software that can be used to create a video tutorial to teach a Math algorithm.

TRK8. Match the following resources with the apps/ software you cand use to create them:

Conceptual maps/ Educational movies/ Electronic presentation/ Interactive activities for practice/ Prezi/ Storyjumper/ Mindmeister/ Twinkl.

Value beliefs (from the following statements, please select the ones that you agree with)

Digital technologies can be used at any stage of the lesson.

Digital technologies can be used in any type of lesson (e.g. learning new skills).

Digital technologies can be used to introduce or practice any mathematical content.

There are digital technologies accessible for any level of severity of intellectual disability.

I think digital technologies allow me to better monitor progress of my students.

Digital technologies motivate and keep students engaged in the task.

Beliefs about the limits of using technologies in the Math education of children with intellectual disabilities

Children with moderate and severe intellectual disabilities do not have the minimum digital skills to use digital technologies effectively.

Children with intellectual disabilities need concrete experiences to learn and digital technologies do not allow them to do this.

Technologies cannot be implemented in the case of children with intellectual disabilities.

Self- efficacy belief in using technology for education

(From strongly disagree- to strongly agree)

SE1. My lessons adequately combine educational approaches with technology and mathematical content.

SE2. I can select technologies that enhance the content of teaching, improve the methods used and the performance of my students.

SE3. I am aware of the limitations of different digital technologies in the learning of mathematics by students with intellectual disabilities through different forms of lesson organization and teaching methods.

SE4. I can select digital technologies that can be easily used by my students with intellectual disabilities to create meaningful experiences for them in learning mathematical operations.

SE5. I continually update my knowledge of new digital technologies in the mathematics education of students with disabilities either through self-study or by attending training courses/workshops etc.

B. Criteria for scoring the answers:

Technology integration practices

P1. What are the types of digital technologies you use in Mathematics activities?

0- none

1- for 1-2 different digital technologies

2- for 3-4 different digital technologies

3- for 5-8 different digital technologies

P2. In general, what would be a mean percent from a lessons's time in which you used digital technology? Please refer to the current year.

0- none

1- less than 10% of the lesson time

2- between 10-40%

3- 50% or more

P3. In most situations, estimate a mean percentage of the taught content in which you used technology. Please refer to the current year.

0- none

1- 10% of the content

2- 30- 50% of the content

3- 75% of the content

P4. During online school I have used the following digital technologies to teach Math.

0- none

1- 1-2 different digital technologies

2- 3-4 different digital technologies

3- 5-7 different digital technologies

P5. When returning from online school to on- site, I have used the following technologies:

0- none

1- 1-2 different digital technologies

2- 3-4 different digital technologies

3- 5-7 different digital technologies

P6. Underneath, you have a list of curricular Math topics that you address with your students with intellectual disabilities. Give examples of digital technologies that you used to teach various content. Include an example. (Math prerequisites/Number concept and numeracy? / Addition, subtraction, multiplication, division/Word problem solving (simple, complex, algorithmic)/Measurement units/Elements of geometry)

We considered the number of indicated digital technologies.

P7. For which teaching function have you predominantly used digital technologies?

Exercise/ Teaching of new concepts/ Assessment/ I do not use it

0- I did not use ie

1- one function

2- two functions

3- all three functions

P8. Select educational digital resources that you use in your Math activities: digital textbook, web pages, encyclopedias, videos.

0-none

1- one resource

2- two indicated resources

3- three indicated resources

4- all four indicated resources

P9. What type of emergent technologies have you used in the classroom?

None/Augmented reality/ Virtual reality/ Robotics/ Other

0- none

1- one type

2- two different types

3- three different types or more

If they selected Other, we attributed point only if the participant named the emergent technology.

P10. From the following educational digital resources, indicate the ones that you most created with available apps: electronic presentations/ virtual panels/ videos, animation, animated strips/ collaborative documents/ conceptual maps/ educational games and interactive exercises/ assessment instruments.

0- none

1 between 1-2 different digital resources

2- between 3 and 4 different digital resources

3- between 5-7 different digital resources

Technology related knowledge items

TRK1. Which technology would you recommend being used in the Math education of children with intellectual disabilities on a large scale?

0- none

1- between one to two different technologies

2- between three to four different technologies

3- between five to eight different technologies

TRK2. Enumerate some advantages of using mobile Math games in the Math education of children with intellectual disabilities.

0- does not give one

1- one advantage

2- between two to three advantages

3- more than three advantages

TRK3. Enumerate some advantages of using interactive PowerPoint in the Math education of children with intellectual disabilities.

0- does not give one

1- one advantage

2- between two to three advantages

3- more than three advantages

TRK4. Underneath, you have possible uses of technology in Math education. Give an example of technology that can support…

TRK4a. the formation of Mathematical representations.

0- does not offer an answer

1- indicates a class of digital technologies

2- indicates an app, soft, platform that has a general content, including Math

3- indicates a digital technology that is specific to Math

TRK4b. discovery learning

0- none

1- a device

2- a class of digital technologies/ resources

3- indicates apps, software etc.

TRK4c. individualization of learning

0- none

1- a device

2- a class of digital technologies/ resources

3- indicates apps, software etc.

TRK4d. independent work and exercise

0- none

1- a device

2- a class of digital technologies/ resources

3- indicates apps, software etc.

TRK5. Do you know technologies of Augmented reality?

1- yes

0- no

TRK6. Give examples of apps/ software that allow you to develop digital resources for Math, such as virtual recapitulative panels.

0- does not offer an answer

1- indicates a class of digital technologies

2- indicates an app, soft, platform that has a general content, including Math

3- indicates a digital technology that is specific to Math

TRK7. Give examples of apps/ software that can be used to create a video tutorial to teach a Math algorithm.

0- does not offer an answer

1- indicates a class of digital technologies

2- indicates an app, soft, platform that has a general content, including Math

3- indicates a digital technology that is specific to Math

TRK8. Match the following resources with the apps/ software you can use to create them:

Conceptual maps/ Educational movies/ Electronic presentation/ Interactive activities for practice/ Prezi/ Storyjumper/ Mindmeister/ Twinkl.

0- no correct matching

1- one correct match

2- two correct matches

3- three correct matches

4- four correct matches

C. **Themes from open- ended questions**

Answers for question TRK 2

Themes	Number
Positive effect on attention	30
Interactivity	13
Effect on motivation	12
Appeal of mobile games	21
Directivity, organization	4
Diversity and variety of learning situations offered through games	5
Repeatability	2
Customization, individualization of the task	2
Well-being, enjoyment	2
Value for learning	2
Other:<br>Support socializing in virtual communities<br>Dexterity, reaction speed<br>Motor skills<br>Accessibility by not requiring writing<br>Development of digital skills<br>Creativity<br>Collaborative work<br>Develops thinking skills<br>Develops language<br>Ease of use and access	 1<br><br>2<br><br>2<br><br>2<br>2<br>3<br>1<br>1<br>1

Answers for question TRK 3

Themes	Number of answers
They are appealing to the student.<br>Draws students' interest	15
Attention	19
Interactivity	11
Immediate feedback	3

Gives a visual support	11
Diverse means of presenting the content (visual- auditory, with various resources)	4
Synthesize essential information, present it in a schematic way	8
Individualization	5
Discovery learning	1
Supports the learning of various Math content	5
